# A Case of Complete Remission of Bilateral Upper Tract Carcinoma In Situ Following Retrograde Bacillus Calmette–Guérin ( BCG ) Instillation via Open‐Ended Ureteral Catheters After Failure Using Double‐J Stents

**DOI:** 10.1002/iju5.70092

**Published:** 2025-09-17

**Authors:** Nanaka Katsurayama, Toshihide Horiuchi, Koichi Nishimura, Kazutaka Nakamura, Takanori Endo, Yuki Nemoto, Nao Nakajima, Daisuke Toki, Tsunenori Kondo

**Affiliations:** ^1^ Department of Urology Tokyo Women's Medical University Adachi Medical Center Tokyo Japan; ^2^ Department of Urology Chiba‐Nishi General Hospital Matsudo City Chiba Prefecture Japan; ^3^ Department of Urology Tokiwakai Jyoban Hospital Fukushima Japan

**Keywords:** bacillus calmette–guérin, carcinoma in situ, kidney pelvis, transitional cell carcinoma, ureteral neoplasm

## Abstract

**Introduction:**

We present a case of carcinoma in situ (CIS) of the bilateral upper urinary tract (UUT) in which long‐term complete remission was achieved through retrograde perfusion of Bacillus Calmette–Guérin (BCG) via open‐ended ureteral catheters following the failure of endoluminal therapy using double‐J stents.

**Case Presentation:**

A 52‐year‐old male patient was diagnosed with CIS of the bilateral UUT and bladder. Intravesical BCG instillation using double‐J stents failed to eradicate persistently positive catheter urine cytology from the bilateral UUT. Since the patient strongly desired kidney‐sparing treatment, retrograde BCG perfusion via open‐ended ureteral catheter was performed. This treatment resulted in durable complete remission lasting 3 years.

**Conclusions:**

Retrograde BCG treatment via double‐J stent may result in suboptimal efficacy due to limited drug exposure to the UUT urothelium. In cases in which kidney‐sparing treatment is required, retrograde BCG perfusion via open‐ended ureteral catheters may be considered a viable therapeutic option.

**Summary**

- Retrograde BCG treatment via intravesical instillation using a double‐J stent is sometimes employed for the management of upper tract urothelial carcinoma in situ. However, this approach may result in suboptimal efficacy due to limited drug exposure to the urothelium of the upper urinary tract. In cases where kidney‐sparing treatment is required, retrograde BCG perfusion via open‐ended ureteral catheters may be considered a viable therapeutic option.

## 1 Introduction

Endoluminal Bacillus Calmette−Guérin (BCG) therapy has been proposed as a kidney‐sparing strategy for primary carcinoma in situ (CIS) of the upper urinary tract (UUT) [1, 2]. Several techniques are available for delivering therapeutic agents to the UUT, including antegrade through nephrostomy and retrograde with a double‐J stent causing vesico‐ureteral reflux (VUR) or with a single‐J stent or open‐ended ureteral catheters. A meta‐analysis reported no significant difference in cancer control among these techniques [3]. However, in vivo porcine model studies showed that retrograde administration with an open‐ended ureteral catheter resulted in higher drug exposure compared to the others [4].

In this case report, we present a patient with carcinoma in situ (CIS) of the bilateral UUT who showed no cytological response to retrograde BCG instillation using double‐J stents. However, long‐term complete remission was achieved following BCG perfusion via open‐ended ureteral catheters.

## 2 Case Presentation

**FIGURE 1:**
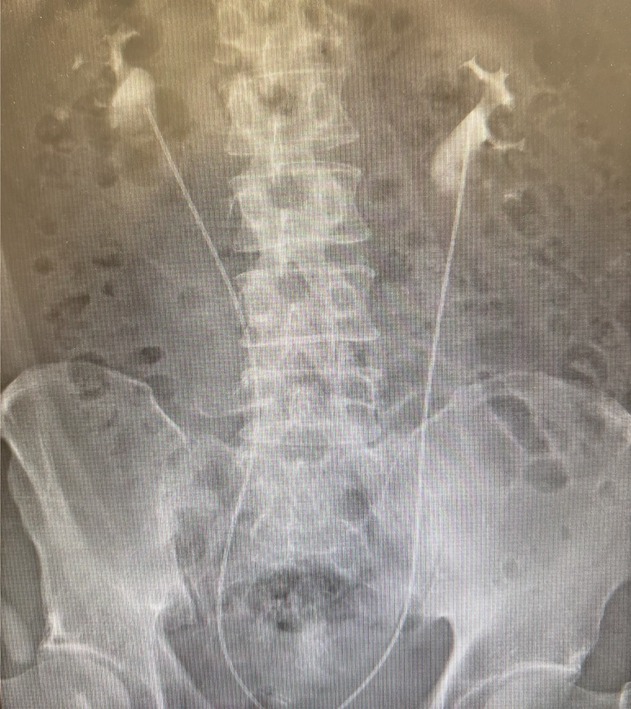
Bilateral placement of open‐ended ureteral catheters into the renal pelvis. Subsequently, Bacillus Calmette–Guérin (BCG) perfusion was initiated.

**FIGURE 2:**
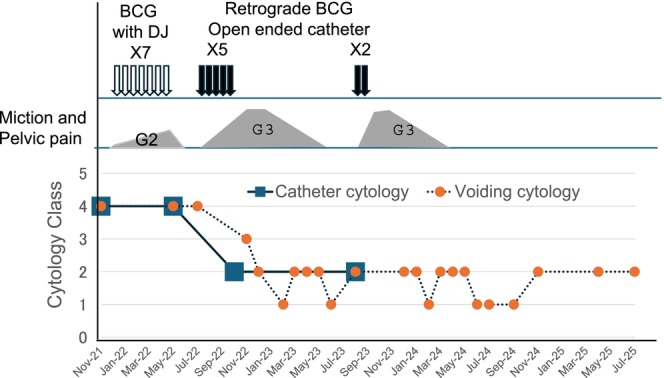
Timeline of treatment and clinical course of the patient.

A 52‐year‐old man visited a local urologist in December 2021 with complaints of residual urine. Urinalysis revealed atypical cells, and the urine cytology was Class IV. Cystoscopy revealed no tumors in the bladder except for some reddish flat lesions, from which biopsy confirmed urothelial CIS. Catheter urine cytology also showed Class IV from the bilateral UUTs, whereas computed tomography and retrograde pyelography showed no tumorous lesions. Thus, the patient was diagnosed with CIS in both the bladder and bilateral UUTs. He underwent seven times weekly intravesical BCG instillations using bilateral double‐J stents from January 2022. Upon placement of the 6 Fr double‐J catheter, VUR to the renal pelvis was confirmed by instillation of 40 mL of contrast medium. BCG (80 mg) dissolved in 40 mL of normal saline was then instilled into the bladder. However, the urine cytology remained persistently positive after BCG therapy. Catheter urine cytology continued to show Class IV throughout the bilateral renal pelvis and ureters, and a random bladder biopsy turned negative. Thus, CIS of bilateral UUT was considered BCG refractory, and total urinary tract exenteration and renal replacement therapy were recommended. He was referred to our hospital for surgery in July 2022. However, he strongly desired kidney‐sparing procedures. We proposed retrograde BCG perfusion via open‐ended ureteral catheters as an alternative to ensure adequate contact of BCG to the UUT urothelium. Upon his consent, this treatment was initiated in August 2022. As no standard BCG instillation protocol has been established yet, we followed previously published methods, as shown in Table 1 [5, 6]. Briefly, open‐ended ureteral catheters were placed into the renal pelvis (Figure 1), and 40 mg BCG dissolved in normal saline was infused into each renal unit over 2 h. After the infusion, both ureteral and Foley catheters were removed. We decided to treat bilateral UUTs simultaneously since a previous study reported no additional toxicity [7]. Fever of 38–39°C resolved within 1–2 days, but micturition pain and urinary frequency required over a week to improve enough to be ready for the subsequent perfusion; instillations were therefore repeated every 2–3 weeks. The patient was admitted to the hospital for every treatment session to monitor for acute adverse events. A timeline of the treatment course is presented in Figure 2. Treatment was discontinued after the fifth instillation due to severe pelvic pain. Catheter urine cytology was negative bilaterally. Pelvic pain, likely due to sacroiliitis associated with BCG‐induced Reiter's syndrome [8], required morphine for symptom control and persisted for over 6 months, while urine cytology remained negative. One year after the initiation of therapy, catheter urine cytology from both UUTs remained negative, and adverse symptoms had completely resolved. In light of the available evidence on high‐risk non‐muscle‐invasive bladder cancer [2] and the patient's strong wishes, we decided to proceed with maintenance therapy in August 2023. However, only two additional instillations could be administered due to recurrent pelvic pain, which required another 6 months to resolve. As of July 2025, voided urine cytology remains negative, and no significant findings were observed on CT scans, suggesting complete remission at 3 years after the treatment.

**TABLE 1:** Protocol for retrograde Bacillus Calmette–Guérin (BCG) perfusion using open‐ended ureteral catheters.

Place 5 Fr open‐ended ureteral catheters into the bilateral renal pelvis under flexible cystoscopy.
2 Tie and fix the bilateral ureteral catheters to an indwelling Foley catheter.
3 Dissolve 40 mg of BCG in 120 mL of normal saline and infuse at a rate of 1 mL/min for 2 h using infusion pumps in each renal unit.
4 Remove both Foley and ureteral catheters after the infusion.
5 Repeat every 2–3 weeks based on the severity and resolution of adverse events.

## 3 Discussion

It has been reported that 84% of patients with UUT‐CIS achieved a complete cytological response to endoluminal BCG instillation, despite the risk of cancer recurrence at 32% and disease progression at 17% [3]. There are three major techniques for delivering BCG to the UUT. The antegrade approach, in which BCG is administered through a nephrostomy, allows for reliable contact with the urothelium [6]. However, this method has disadvantages such as tumor seeding along the nephrostomy tube, drug leakage, and reduced quality of life due to the nephrostomy itself [9]. Retrograde administration using a double‐J stent is often utilized because of its relative simplicity [10]. In this method, an indwelling stent induces artificial VUR, allowing intravesically instilled BCG to be delivered to the UUT. Korke et al. investigated the efficiency of VUR induction and showed that the occurrence of VUR was associated with volumes instilled in the bladder [11]. It should be noted that VUR was confirmed only at 63%, even if 360 mL was instilled into the bladder.

The third technique is retrograde instillation via an open‐ended ureteral catheter, first reported by Sharpe et al., who demonstrated a complete cytological remission rate of 73% [7]. Although repeated retrograde catheter insertion is required at the time of drug administration, it enables accurate and reliable BCG delivery to the UUT. Liu et al. compared the efficiency of drug contact with the UUT urothelium among the three delivery methods using porcine models [4]. This study has shown that retrograde infusion via open‐ended ureteral catheter, as employed in this case, resulted in the highest drug delivery efficiency, suggesting that this technique may be optimal for drug delivery to the UUT. However, according to a review article based on real‐world experience, there is currently no evidence supporting the superiority of any single delivery method [9]. Therefore, the applicability of the experimental findings to clinical practice needs to be determined.

In conclusion, we report a case of CIS of the UUT in which long‐term complete remission was achieved using retrograde BCG instillation via open‐ended ureteral catheters after failure of treatment using double‐J stents. The delivery technique should be selected by considering the efficiency of drug contact with the urothelium in the UUT, as well as the invasiveness of each technique.

## Disclosure

Approval of the Research Protocol by an Institutional Review Board: Not applicable.

Research Registration: Not applicable.

## Consent

Written informed consent for publication was obtained from the patient.

## Conflicts of Interest

Tsunenori Kondo received honoraria from Takeda Pharmaceutical, MSD, and Eisai.
